# Enhancing the Implementation of the Virtual Pediatric Trauma Center Using Practical, Robust, Implementation and Sustainability Model: A Mixed-Methods Study

**DOI:** 10.1089/tmr.2022.0020

**Published:** 2022-07-25

**Authors:** Jennifer L. Rosenthal, Sarah C. Haynes, Bethney Bonilla, Katherine Rominger, Jacob Williams, April Sanders, Raynald A. Orqueza Dizon, Kendra L. Grether-Jones, James P. Marcin, Michelle Y. Hamline

**Affiliations:** ^1^ Department of Pediatrics, University of California Davis, Sacramento, California, USA.; ^2^ Center for Healthcare Policy and Research, University of California Davis, Sacramento, California, USA.; ^3^ Department of Emergency Medicine, University of California Davis, Sacramento, California, USA.

**Keywords:** child, patient transfers, communication implementation, science, telemedicine, trauma centers

## Abstract

**Background:**

This article describes factors related to adoption, implementation, and effectiveness of the Virtual Pediatric Trauma Center intervention, which uses telehealth for trauma specialist consultations for seriously injured children. We aimed at (1) measuring RE-AIM (Reach, Effectiveness, Adoption, Implementation, Maintenance) implementation outcomes and (2) identifying PRISM (Practical, Robust, Implementation, and Sustainability Model) contextual factors that influenced the implementation outcomes.

**Methods:**

This interim implementation evaluation of our telehealth trial used a convergent mixed-methods design. The quantitative component was a cross-sectional analysis of pediatric trauma encounters using electronic health records. The qualitative component was a thematic analysis of written and verbal feedback from providers and family advisory board meetings. We compared the quantitative and qualitative data by synthesizing them in a joint display table, organized by RE-AIM dimensions. We categorized these key findings into the PRISM domains.

**Results:**

During the first 10 months of this trial, 246 subjects were randomized, with 177 assigned to standard care and 69 assigned to telehealth. Four referring sites transitioned from standard care into their intervention period. PRISM contextual factors that influenced RE-AIM implementation outcomes included the following findings: Providers struggle to remember, interpret, and navigate intervention workflows; providers have preconceived ideas about the intervention purpose; the intervention mitigates parents' anxieties about the transfer process.

**Discussion:**

This study revealed implementation challenges that influence the overall success of this telehealth trial. Early identification of these challenges allows our team the opportunity to address them now to optimize the intervention reach, adoption, and implementation. This early action will ultimately enhance the success of our trial and the ability of our intervention to achieve broad impact.

## Introduction

Seriously injured children often require transfer to regional Level I pediatric trauma centers to receive definitive care.^1,2^ This transfer process is usually facilitated via a telephone consultation from a trauma center provider to remotely assist the referring provider. This standard-of-care practice, however, has limitations in that the remote trauma center provider communicates only with the referring provider and thus does not communicate with the parent or guardian (referred to as “parent” hereafter) before transfer. To better improve family-centered communication with the family, we developed a new model of care, the “Virtual Pediatric Trauma Center” (VPTC), which uses telehealth to virtually bring the trauma center provider to the patient's bedside.

A clinical trial to compare the VPTC with the current standard of pediatric trauma care is ongoing (ClinicalTrials.gov NCT04469036). In designing this trial, we hypothesized that the VPTC intervention would enhance parent support and understanding, which will ultimately improve family-centered outcomes. The outcomes of interest include the parent experience of care, parent distress, health care utilization, and out-of-pocket cost burden.

The mechanisms by which the VPTC is hypothesized to impact the trial's family-centered outcomes are based on lessons learned from family advisory board meetings and parent interviews during the trial planning phase. The mechanisms include two main mediators in the causal pathway. The first mediator is that trauma center providers enhance family-centered care via remote support to parents. This parental support positively impacts the parent experience of care and parent distress outcomes. The second mediator is that care is enhanced via remote information sharing to enhance parental understanding.

The content of the information sharing is based on the parents' real-time needs and can include topics ranging from the child's clinical management to the logistics of the transfer process. Improved understanding strengthens parent activation and ultimately impacts the health care utilization and out-of-pocket cost burden outcomes. Improved understanding also impacts parent experience and parent distress.

In addition to examining VPTC effectiveness, we must also consider its implementation, diffusion, and sustainability.^3^ Early attention to dissemination and implementation is needed to overcome the traditional focus of internal over external validity to close the translation gaps between research and practice. Dissemination and implementation frameworks can be applied throughout all phases of a program, from intervention planning through dissemination.

Applying a framework to conduct an interim evaluation during a trial is a strategy that can permit early identification of implementation challenges so that adjustments to systems and processes can be made sooner rather than later.^4^ Early assessment and adjustment can be crucial; for example, interventions with poor adoption will not be effective.^5^ Evaluations focused on contextual factors (i.e., any elements that are not part of the intervention itself) are critical to optimizing an intervention's success in achieving long-term and broad impact.^6^

The Practical, Robust, Implementation and Sustainability Model (PRISM)^5^ is an implementation model designed to help conceptualize and understand the contextual conditions that influence implementation success. PRISM focuses on the following domains: Intervention (Organizational perspective, Patient perspective), Recipients of the intervention (Organizational characteristics, Patient characteristics), Implementation and sustainability infrastructure, and External environment.^5^ PRISM measures implementation success using the RE-AIM (Reach, Effectiveness, Adoption, Implementation, Maintenance)^7,8^ outcomes; therefore, implementation adjustments should be guided by RE-AIM measurements.

The purpose of this article is to describe factors related to the adoption, implementation, and effectiveness of a novel telehealth model of family-centered care for pediatric traumas. This article presents our use of PRISM to explore mechanisms that influence implementation success of the VPTC. Specifically, we aimed at (a) measuring RE-AIM implementation outcomes and (b) identifying PRISM contextual factors that influenced the implementation outcomes.

## Methods

### Design

This intervention evaluation was a mixed-methods study using a convergent design. The rationale for a convergent design was to combine and compare the quantitative and qualitative data with the intent of obtaining a comprehensive understanding of the contextual factors that influenced outcomes within each RE-AIM dimension. Our study was guided by the PRISM domains.^5^

The quantitative component was a cross-sectional analysis of pediatric trauma encounters using electronic health records. The qualitative component was a thematic analysis of written and verbal feedback from providers and family advisory board meeting agendas and minutes. The quantitative and qualitative data collection and analyses were conducted separately and independently from each other. Once the two sets of initial results were completed, we merged the results of the two data sets and interpreted the combined results. The University of California Davis Institutional Review Board approved this study. This article follows the Standards for Reporting Implementation (StaRI) studies.^9^

### Setting

The hospital that provides the pediatric trauma consultations to the referring emergency departments (EDs) is a 121-bed quaternary care children's hospital in Northern California. This hospital is the only Level I pediatric trauma center in the region, serving as the referral center for more than 1 million children across a 33-county region covering 65,000 square miles.^10^ This hospital receives transfers from 130 different hospitals and EDs across the region, accepting ∼2500 pediatric transfers annually. Approximately 700 pediatric trauma consultations occur annually; an additional ∼750 seriously injured children present directly to the trauma center's ED.

### Intervention and patient population

This interim evaluation was conducted for a clinical trial with a prospective stepped-wedge trial design.^11^ On November 30, 2020, all referring hospital sites began in the standard-of-care condition (no use of telehealth). Every 8 weeks, one site transitioned into the VPTC intervention period. The order of this site-level transition is based on stratified random assignment.

Eligible trial subjects include all pediatric trauma patients aged younger than or equal to 17 years who present to 1 of the 11 participating referring EDs with an acute traumatic injury. The subject must have a transfer consultation call to one of the following services at the trauma center: trauma surgery, orthopedic surgery, or neurosurgery. Eligible subjects must have a parent or guardian present with them at the referring facility. Subjects who are wards of the state, receive cardiopulmonary resuscitation, or die in the ED are excluded.

For the VPTC intervention, after an eligible injured child arrives to a referring site and the referring physician wishes to discuss a potential transfer with a trauma center provider, a telephone call is initiated through the receiving Level I pediatric trauma center's transfer center. The trauma surgeon joins this call for a brief consultation. The referring provider, transfer center nurse, and trauma center provider are instructed to remind one another when a pediatric trauma encounter is eligible to receive the VPTC intervention.

The referring site then places the telehealth cart at the patient's bedside; this cart is a computer with an omnidirectional microphone, speaker, and pan-tilt-zoom camera, mounted on a hospital grade pole with wheels. A consulting provider from the pediatric trauma center then initiates a telehealth connection at the bedside with the parent(s). The computer at the referring site automatically responds, and the virtual consultation proceeds using a commercially available videoconferencing software. We use two different videoconferencing platforms for telehealth (Zoom, San Jose, CA, USA and Teledoc, Purchase, NY, USA) to accommodate referring sites' preferred platforms.

### Implementation approach

We convened a multidisciplinary implementation team to monitor study metrics throughout the trial and respond to any implementation issues. Metrics reviewed by the team on a weekly basis include trial enrollment, protocol adherence, and survey response rates. The 13-member multidisciplinary implementation team includes a trauma surgeon, trauma surgery nurse practitioner, pediatric surgeon, pediatric emergency medicine physician, transfer center nurse, referring ED pediatric medical director, telehealth medical director, project managers, stakeholder engagement expert, and research assistants. The parent team member has a child who experienced an injury that required transfer to the trauma center. We additionally have a 12-member family advisory board that consists of parents of children who experienced a traumatic injury.

The pediatric trauma center provider designated to initiate this telehealth connection is determined based on workflows that account for type of trauma (e.g., isolated orthopedic injury) and surgeon availability. Trauma surgery nurse practitioners are available to conduct telehealth from 6 AM to 1 AM. Since the trauma surgery nurse practitioners are not part of the transfer consultation for standard-of-care encounters, the transfer center sends a separate page to notify the trauma surgery nurse practitioners when a VPTC subject is eligible for a telehealth consultation. From 1 AM to 6 AM, the trauma surgeon initiates the telehealth consultation. Pediatric orthopedic surgeons initiate the telehealth consultation in specific cases when an orthopedic consultation is requested by the trauma surgeon or the referring hospital.

A copy of the VPTC protocol is in the transfer center office for the transfer center nurses to reference; it includes the list of ED sites in the intervention period. All 47 VPTC pediatric trauma center providers were trained on the VPTC protocol and associated telehealth; these providers included 20 trauma surgeons, 21 trauma surgery nurse practitioners, 4 pediatric orthopedists, and 2 pediatric neurosurgeons. Trainings were conducted both individually and at division meetings. Each referring site has a VPTC champion who helps coordinate a virtual group training with ED providers before the transition into their intervention period. Site champions are asked to disseminate information about the VPTC trial to additional providers not present at the virtual training. Regular e-mail check-ins with both referring and accepting providers after each eligible patient serve as frequent reminders of the VPTC intervention.

### Data sources and analysis

Ongoing implementation feedback is obtained in a variety of ways. A research assistant conducts audits every weekday from a pediatric trauma report generated from the electronic health record. The research assistant solicits feedback via e-mail to the consulting providers following each eligible pediatric trauma encounter assigned to the intervention. The e-mail asks questions about the provider's overall experience using or not using telehealth for that encounter, suggestions for improvements, perceived intervention value, and technical issues. The e-mail reminds providers to contact the study team at any time to report and troubleshoot issues related to using the intervention. Finally, we update our family advisory board on implementation progress and solicit their feedback during quarterly meetings.

#### Electronic health record data

We obtained patient-level data from the electronic health record for each of the eligible trial subjects. Patient characteristics included age, race, ethnicity, preferred language, and insurance. Patient clinical variables included presenting injury, chief complaint, date and time of consultation, and disposition. To measure intervention reach, adoption, and implementation, we used VPTC trial administrative documents to obtain trial inclusion, exclusion, randomization, and telehealth usage data, including intervention usage over time (run charts) and reasons for protocol deviations.

We conducted descriptive analyses for each variable of interest. We calculated the proportion of encounters with intervention use as a ratio of the number of eligible pediatric trauma encounters for whom the VPTC intervention was used (numerator) to the total number of encounters assigned to the intervention (denominator).

#### Stakeholder feedback

We obtained all e-mail exchanges addressing intervention feedback between implementation team members and users of the intervention. We also included data from an interview conducted following an e-mail exchange with a provider who wanted to provide more thorough feedback verbally. This interview was audio recorded and professionally transcribed. The transcription was reviewed for accuracy by the interviewer. In addition, we included all meeting agendas and minutes recorded from quarterly family advisory board meetings.

The family advisory board was convened before trial onset and, thus far, has met virtually due to pandemic restrictions. During the meetings, a research team member took real-time participatory notes, which were documented on the meeting agendas; thus, the final documents included both meeting agendas and minutes.

Qualitative data were analyzed using thematic analysis^12,13^ with the framework approach.^14^ The framework consisted of the five RE-AIM dimensions. Eight team members independently conducted analytic memo writing and open coding of the documents to identify potential findings that did not fit into the *a priori* framework categories. These researchers agreed that the framework categories adequately reflected the prominent findings in the data. We subsequently independently performed memo writing and coding of the documents using the *a priori* codes related to the RE-AIM dimensions.

**FIG. 1.**
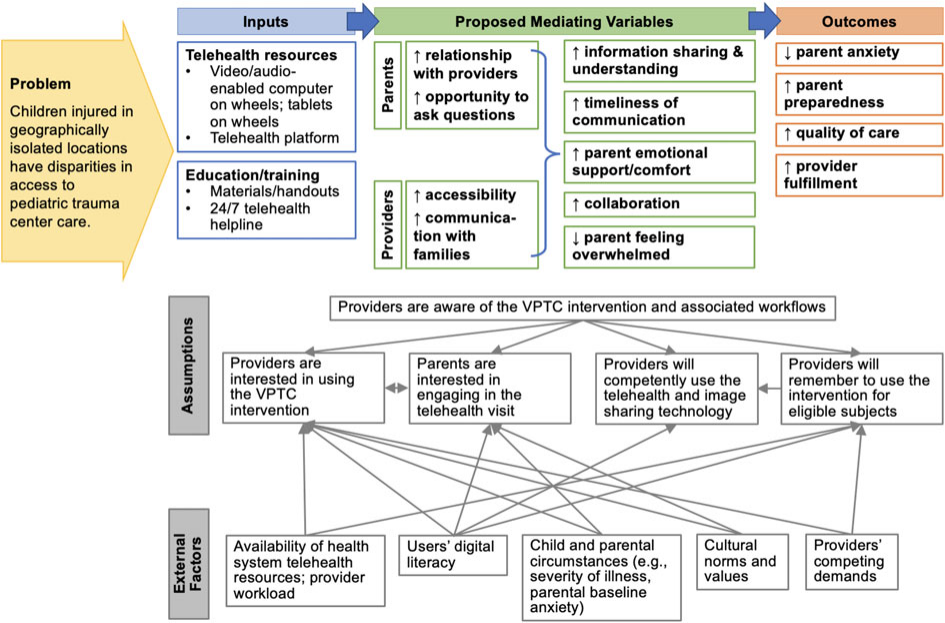
Logic model of the relationships among the intervention's activities and its outcomes. VPTC, virtual pediatric trauma center.

We met as a team to ensure consensus on application of codes, develop tentative categories, and examine the data for patterns and variations. We created a participants' role-ordered matrix to explore how variables related to participants' role types. We developed tentative hypotheses about relationships among categories and searched for negative and qualifying evidence. The recurrent unifying concepts and identified linkages and patterns between the categories became analytic themes. We used the themes to create a logic model of the relationships among the VPTC intervention's activities and its outcomes (Fig. 1). We used ATLAS.ti to organize and store coding and data analysis.^15^

#### Integration

We compared the results of the quantitative and qualitative data by synthesizing them in a joint display table, organized by the RE-AIM dimensions. We identified to what extent and in what ways the two sets of results (quantitative and qualitative) converged, diverged, or related to each other. We categorized these key findings into the PRISM domains. We used a narrative discussion to report our integrated findings.^16^

## Results

During the first 10 months of the ongoing 24-month trial, 276 pediatric trauma subjects were assessed for eligibility, of whom 17 subjects did not meet inclusion criteria. Thirteen (4.7%) subjects were excluded for the following reasons: no parent/guardian present (*n* = 4), ward of the state (*n* = 6), and death before transfer or active cardiopulmonary resuscitation at the time of transfer consultation (*n* = 3).

Overall, 246 subjects were randomized, with 177 assigned to standard of care and 69 assigned to the VPTC intervention. Four referring sites transitioned from standard of care into their intervention period. The joint display table of quantitative and qualitative RE-AIM outcomes is shown in Table 1. Table 2 presents key findings (elements) for each PRISM domain, including the RE-AIM outcomes affected by the represented elements. The following sections present our integrated results in passage format organized by theme.

**Table 1.** Joint Display of Quantitative and Qualitative Reach, Effectiveness, Adoption, Implementation, and Maintenance Outcomes

Dimension	Qualitative data	Quantitative data	Mixed-methods comparison
Reach	• Some providers do not use VPTC for certain circumstances (e.g., severe trauma) due to their perception that telehealth is disadvantageous in such contexts	• Assessed for eligibility (*n* = 276) • Did not meet inclusion criteria (*n* = 17, 6.2%) • Excluded (*n* = 13, 4.7%); reasons include ward of state (*n* = 6), no guardian present (*n* = 4), active CPR, or death before transfer (*n* = 3) • Among intervention arm subjects (*n* = 69), adjusted analysis found that use of VPTC did not vary by patient age, race, ethnicity, or insurance type	• Perception that VPTC is disadvantageous for certain circumstances such as severe trauma is discordant with the intended target population that includes seriously injured children
Effectiveness	• VPTC is perceived [mostly by trauma nurse practitioners] to reduce parental anxiety by enhancing parental preparedness	• ^a^Comparison of impact of VPTC versus standard of care on outcomes will be reported in final evaluation; outcomes include parent experience of care, parent distress, health care utilization, and out-of-pocket cost burden	• Perception that VPTC reduces parental anxiety aligns with outcomes of interest, including parent experience of care and parent distress • Perception that VPTC enhances parental preparedness aligns with outcomes of interest, including health care utilization and cost burden
Adoption	• Adoption of VPTC by some referring sites is impeded by existing technologies conflicting with the newly introduced intervention	• Site-level telehealth intervention adherence: ^b^Site A: 15/25 (60%), Site B: 1/1 (100%), Site C: 10/33 (30%), and Site D: 0/10 (0%)	• Qualitative and quantitative data are congruent in that sites have variable success in adopting VPTC
Implementation	• VPTC is not always being delivered as intended, because implementation workflows rely on individuals to remember, interpret, and navigate the process	• The most common reasons for not using telehealth when eligible: forgetting about VPTC (37%), referring site declined (14%), unavailable trauma center provider (14%), protocol misinterpretation (7%), and technology issues (5%) • Telehealth intervention adherence^b^ by provider type: nurse practitioners 41%, pediatric orthopedists 29%, trauma surgeons 0%, and neurosurgeons 0%	• Qualitative and quantitative data are congruent in that lack of awareness (i.e., forgetting) is a major barrier to VPTC implementation • Trauma surgery nurse practitioners have the highest telehealth adherence proportion, despite missed opportunities due to the trauma center nurse forgetting to notify the nurse practitioner of eligible encounters (whereas surgeons are automatically notified of all pediatric trauma encounters)
Maintenance	• Long-term delivery of the VPTC will be supported by providers amassing positive experiences with using the intervention, specifically witnessing the benefits of enhanced parental support	• ^a^Post-trial intervention outcomes will be reported in the final evaluation	

^a^ Planned implementation outcome measurement for the future evaluation stage.

^b^ Number of eligible encounters that used telehealth divided by the number of eligible intervention-arm encounters.

CPR, cardiopulmonary resuscitation; VPTC, Virtual Pediatric Trauma Center.

**Table 2.** Practical, Robust, Implementation and Sustainability Model Domain Key Findings

PRISM domain	Key findings	RE-AIM outcomes affected
Intervention		
Organizational perspective	• Some providers struggle to remember, interpret, and navigate the VPTC workflows • Referring providers lack awareness of VPTC and struggle to learn in real time • Providers have preconceived ideas about the purpose of telehealth • Desire to initiate telehealth will increase over time as providers amass positive experiences reinforcing its benefits to parents	Reach Effectiveness Adoption Implementation Maintenance
Patient perspective	• VPTC is patient/parent-centered	Effectiveness Adoption
Recipients		
Organizational characteristics	• Trauma nurse practitioners initiate most telehealth consultations and, thus, experience the benefits of VPTC enhancing parent support • Providing remote clinical recommendations is beyond the scope of practice for the trauma surgery nurse practitioners	Effectiveness Adoption Implementation Maintenance
Patient characteristics	• Among VPTC intervention-arm subjects, mean (SD) age is 7 (5) years, 61% have public insurance, 37% are White, 39% are Hispanic or Latinx, and 91% are English speaking • Parents of injured children are anxious due to not knowing what to expect during/after transfer to the trauma center	Reach Effectiveness
Implementation and sustainability infrastructure	• Automated systems are needed to remind providers to use VPTC • Parents can only receive the VPTC if providers initiate the telehealth consultation	Reach Effectiveness Adoption Implementation Maintenance
External environment	• Reimbursement for telehealth consultations is perceived by trauma center providers to be relatively small and thus not a driver of VPTC use	Effectiveness Adoption Implementation Maintenance

PRISM, Practical, Robust, Implementation and Sustainability Model; RE-AIM, Reach, Effectiveness, Adoption, Implementation, Maintenance; SD, standard deviation.

### Reach, adoption, and effectiveness of the VPTC intervention are hindered by implementation workflows that rely on individuals to remember, interpret, and navigate the intervention process

Among the 69 eligible pediatric trauma encounters assigned to the intervention, 26 (37.7%) of them appropriately received a telehealth consultation. Unintentional protocol deviations represented most of the reasons for not using telehealth when eligible. To date, the most common reasons for not using telehealth when eligible included forgetting about the VPTC intervention (37.2%), referring site declined (14.0%), unavailable trauma center provider (14.0%), protocol misinterpretation (7.0%), and technology issues (4.6%).

Depending on the unique circumstances of each individual trauma encounter, different workflows exist that determine which provider will perform the telehealth consultation and the timing of initiating that connection. Since each encounter requires individuals to interpret the situation, automation is not built into the VPTC workflows. By relying on the actions and judgments of individuals, VPTC users are deviating from the study protocol and not using telehealth for eligible intervention-group subjects.

Some of this deviation is unintentional, whereby users are unaware of protocol details. One trauma nurse practitioner wrote, “The patient was an ortho admit transfer, so there was some issues with the trauma NP roles and doing the VPTC. Are we only doing it for trauma transfers? … What is the role for the trauma NP's for other services?” Another trauma surgeon understood the protocol but shared, “To be honest, I forgot that ortho had to review stuff first.”

Other users misinterpreted the protocol despite reading it in real time. As one pediatric orthopedist wrote, “I asked our transfer center if we should make this a VPTC patient and was told they thought it was just for patients being transferred. He even read me the sheet they had up in the transfer center in regards to the study and was not sure how to interpret.”

Some of the intervention deviation is unintentional, whereas other deviation is intentional. Referring or consulting providers sometimes decide that telehealth would be disadvantageous, despite other clinical providers thinking that use of telehealth would be beneficial. One trauma surgeon wrote, “The [referring site] doc specifically asked to skip the study intervention because the kid was very acute (pulseless extremity) and they wanted to expedite transfer (appropriately).” In response to this decision, the referring hospital's site champion wrote: “I wonder, if we debriefed the physicians involved on both sides, if that was the right choice or not. … I think it's going to take some time for people to get familiar with the process and recognize its utility.”

### Implementation of the VPTC intervention across multiple referring sites requires attention to their unique needs and circumstances

Each referring site has unique contextual factors influencing the success of implementation. For example, some sites have existing technologies and workflows that do not intuitively merge with the newly introduced VPTC intervention. One referring provider wrote about their challenges with VPTC, highlighting that they already use a telehealth cart for stroke consultations and they were uncertain of whether or not that cart should also be used for VPTC: “We brought our telemedicine robot that we typically utilize for strokes into the resuscitation room, but we were unsure if UCD knew how to access that particular robot.”

Other contextual factors at referring sites that have hindered implementation include being overcrowded and lacking physical space for the telehealth cart: “I don't know that I would have the physical room for the robot. Poor kiddo never got out of the lobby.”

Awareness of the VPTC trial is not reaching all referring site providers. Real-time guidance by the trauma center providers cannot consistently overcome this lack of awareness and training. One trauma center provider explained, “The ER doctor did not know how. … Nor did she know about the study.” Some referring providers cannot navigate the VPTC intervention without training, whereas other providers can successfully use telehealth as intended by locating the telehealth cart and rolling it to the patient's bedside.

One trauma surgeon wrote, “The referring physician seemed unfamiliar with the process, but seemed to work it out easily at her facility and there did not seem to be any delay.” Despite these successful connections, some trauma center providers shared dissatisfaction with encounters whereby the family was not told that a telehealth consultation would occur or the telehealth cart was not placed in the patient's room.

Regarding site-level intervention adoption, the first site to transition into the intervention period (Site A) had 25 intervention-group subjects, of whom 15 (60.0%) received telehealth. The second site to transition (Site B) had 1 subject; this subject received telehealth. Site C had 33 intervention-group subjects, of whom 10 (30.3%) received telehealth. The most recent site to transition (Site D) has had 10 intervention-group subjects, of whom none received telehealth. Compared with the other three sites, Site D has the greatest annual ED visit volume and the highest annual number of pediatric trauma transfers to the trauma center. Forgetting about the VPTC intervention was the reason given for deviating from the protocol for 70.0% of the Site D encounters.

### Preconceived ideas about the purpose of telehealth presents challenges to implementation, which impedes broad adoption

The VPTC was designed to improve family-centered outcomes by enhancing parent support and parent understanding. This focus on parent support and understanding differs from our typical use of telehealth consultations. Historically, our children's hospital has used telehealth consultations to provide remote clinical decision making to referring providers. This paradigm shift to focus on parent support and understanding presents challenges, because the providers have preconceived ideas that the purpose of a telehealth consultation is for clinical decision making. Such preconceived ideas do not align with the VPTC.

Although remote support to parents is possible with every telehealth consultation, clinical decision making is only possible when a physician (not a nurse practitioner) initiates the telehealth consultation. Providing clinical recommendations is beyond the scope of practice for the trauma surgery nurse practitioners.

Among the 26 telehealth consultations, trauma surgery nurse practitioners—whose scope of practice excludes providing remote clinical recommendations—initiated 22 (84.6%) of the consultations. Pediatric orthopedists initiated 4 (15.4%) consultations; trauma surgeons and neurosurgeons initiated none of the consultations.

This distinction that clinical decision making is not supported when nurse practitioners initiate the telehealth consultation confuses users at both the referring sites and the trauma center. One referring provider demonstrated their preconceived ideas about telehealth and explained the discordant understanding of the purpose of telehealth for an encounter that ultimately did not use the intervention: “The trauma physician I spoke to made it seem to me that the telemedicine consult is only to give patients/families further understanding, expectations and increased satisfaction of the trauma transfer as opposed to the utilizing it to decide to transfer. I thought this child was a perfect case as he has facial fractures that are not always surgical and therefore, we could have used the telemedicine as a decision point on transfer vs not. In the end, we decided to transfer on the phone consult without using the [telehealth] system.”

Providers' preconceived ideas about telehealth consultations and their ensuing confusion are impacting satisfaction among the users. One trauma surgery nurse practitioner shared, “I think the purpose keeps getting lost. This isn't a clinical consultation … I just hope that through time that these providers or the hospitals will fully grasp the purpose of the study. That's my big thing.”

### Adoption of the VPTC will increase over time as comfort with using the technology increases and as providers amass positive experiences reinforcing its benefits

Some trauma center providers expressed discomfort with initially using telehealth. However, their uneasiness with the technology waned over time. One trauma surgery nurse practitioner described her evolving experience: “[Telehealth] took me out of my comfort zone because I usually don't like speaking in front of people. My first phone call. … I was so embarrassed. I was talking really fast, and I don't think that any of us, both sides, got anything out of it. … But as time went by and I did more of the video visits, getting more used to it, I got more comfortable in the role. … It took some getting used to trying to figure myself out in this.”

Multiple trauma center providers expressed that telehealth use for pediatric traumas is particularly beneficial to families. Specifically, telehealth is perceived to reduce parental anxiety by enhancing parental preparedness. As one trauma surgery nurse practitioner shared, “This last one went wonderfully! The parents recognized me on arrival and both smiled upon seeing a familiar face. … Mom said that she appreciated the video visit because she knew what to expect and that eased her anxiety.”

Another nurse practitioner shared a similar experience and how these positive experiences reinforce use of the intervention: “I told the family member, ‘I will see you when you get here.’ When the mother walked in, she was wide-eyed, and she was so overwhelmed with so many people. Then we locked eyes, and she smiled. She told me toward the end, she said, ‘I was really overwhelmed, and I'm usually anxious, but I saw you, and it just made the experience better.’ Yes, that was really heartwarming for me. It reinforces why this needs to be done.”

These perceived telehealth benefits were almost exclusively expressed by trauma surgery nurse practitioners. Their positive perceptions of telehealth, and thus increased buy-in to use the intervention, help them to overcome implementation barriers. Nurse practitioners explained how they have initiated telehealth even when leaving the hospital at the end of a shift: “Literally I was walking out of the building.” The trauma surgeons, pediatric orthopedists, and referring providers infrequently shared the benefits of the VPTC intervention; these users mostly focused on implementation challenges and questioned the effectiveness of telehealth for pediatric trauma consultations.

Among the 69 pediatric trauma encounters assigned to receive the intervention, the VPTC workflows designated the trauma surgery nurse practitioner to initiate 49 (71.0%) of the telehealth consultations. The pediatric orthopedist was designated for 14 (20.3%) of the encounters; the trauma surgeon was designated for 6 (8.7%) of the encounters. Regarding adherence to initiating telehealth when designated to do so, the adherence rates by provider type were 40.8% for nurse practitioners, 28.6% for pediatric orthopedists, and 0% for trauma surgeons. The nurse practitioner initiated two of the telehealth consultations designated to the pediatric orthopedist.

## Discussion

This PRISM evaluation reports our findings of a mixed-methods study about the contextual factors that influence RE-AIM dimension outcomes. We conducted this interim evaluation early in the clinical trial to identify and mitigate potential implementation issues. The qualitative phase identified several factors impeding broad adoption and effectiveness of the VPTC intervention, including complicated workflows that are not automated, unique needs across different sites, and preconceived ideas about the intervention purpose. However, once providers use the intervention and amass positive experiences that reinforce the effectiveness of telehealth in improving parental preparedness and reducing parental anxiety, these providers gain intervention buy-in.

This buy-in is a facilitator to intervention adoption; users with buy-in are willing to overcome the implementation challenges. The quantitative phase results converged with these qualitative results. Indeed, trauma surgery nurse practitioners are initiating most of the telehealth consultations and therefore witnessing firsthand the benefits of the VPTC intervention. The nurse practitioners had the highest adherence rate to initiating telehealth when designated to do so. Figure 1 presents a logic model of the relationships among the VPTC intervention's activities and its outcomes. As shown in the logic model, multiple external factors influence the assumptions about the VPTC intervention.

Findings from our intervention evaluation were presented to the research team, and a plan was activated to begin addressing the identified implementation challenges. We presented the preliminary plan to our family advisory board and worked with them to refine our strategies. First, we will simplify the implementation workflows. One strategy to consider is to have the trauma surgery nurse practitioners initiate all telehealth consultations. By removing alternative workflows that designate different timings and providers to initiate telehealth, there will be one path that is easier for users to understand.

Second, we will explicitly message that the purpose of telehealth use in the VPTC is to enhance parental support and understanding. Remote clinical recommendations might still occur (e.g., when a surgeon rather than a nurse practitioner initiates a telehealth consultation), but we will send a message to all users that remote clinical decision making is not an expectation with VPTC. Third, we will assist referring sites with incorporating the VPTC intervention into their existing workflows. We will hold additional meetings to engage referring providers in identifying local needs and solutions.

Fourth, to reinforce the benefits of the VPTC intervention and thus promote buy-in, we will collect and disseminate parent testimonials on their experiences with telehealth. Finally, we will work with the transfer center to identify strategies to help them remember the VPTC trial. We will discuss potential ways to automate the process of notifying the trauma surgery nurse practitioners of a VPTC intervention subject.

Despite the promise of VPTC to enhance family-centered care delivery for seriously injured children, the effectiveness of telehealth is limited unless we can increase intervention adoption. Our telehealth implementation challenges are consistent with prior research. Telehealth technology learning barriers have been reported extensively in the literature.^17–19^ In addition, efforts to encourage providers to use telehealth have been described in prior publications.^20,21^

The novel coronavirus pandemic vastly increased telehealth use for service lines such as ambulatory care visits.^22–26^However, the use of telehealth for trauma consultations is application of telehealth in a vastly different setting. Providers in both the referring EDs and the trauma center have competing demands, and trauma encounters are inherently unscheduled events that can occur when providers are unavailable. These differences highlight how contextual factors differ based on the unique setting in which an intervention is being delivered. Importantly, context is also dynamic; thus, adaptations to interventions must be ongoing and iterative.^6^

The use of mixed-methods and application of PRISM are strengths of this study. Limitations include being conducted at a single telemedicine program at a children's hospital. Findings from this evaluation may not be generalizable to other hospitals, as contextual factors are likely unique to our setting. Although we include only one trauma center, this evaluation included four referring ED sites. Ultimately, the VPTC trial will include eleven referring sites. Another limitation is that our multidisciplinary implementation team includes only one parent. However, we also presented the findings of our evaluation to our family advisory board to obtain their feedback on adapting our implementation strategies. Further, there are additional unknown and unmeasured factors that influenced the implementation of the VPTC intervention. Despite these limitations, this evaluation enhanced our understanding of factors related to implementation outcomes of our pediatric trauma telehealth intervention. Our use of PRISM to conduct an evaluation of a telehealth intervention can be used as an example for other telehealth researchers and providers wanting to enhance their local program implementation.

## Conclusions

The mixed methods findings from this PRISM evaluation revealed implementation challenges that influence the overall success of the VPTC trial. Conducting this evaluation early in the trial process allowed the research team to identify areas of intervention implementation needing improvement. Addressing these challenges now will ultimately enhance the ability of VPTC to achieve broad impact.

## Abbreviations Used

CPR: cardiopulmonary resuscitation
ED: emergency department
PRISM: Practical, Robust, Implementation and Sustainability Model
RE-AIM: Reach, Effectiveness, Adoption, Implementation, Maintenance
VPTC: Virtual Pediatric Trauma Center
